# The Influence of 3′UTRs on MicroRNA Function Inferred from Human SNP Data

**DOI:** 10.1155/2011/910769

**Published:** 2011-10-25

**Authors:** Zihua Hu, Andrew E. Bruno

**Affiliations:** ^1^Center for Computational Research, New York State Center of Excellence in Bioinformatics and Life Sciences, Departments of Ophthalmology, Biostatistics, and Medicine, State University of New York (SUNY), Buffalo, NY 14260, USA; ^2^SUNY Eye Institute, Syracuse, NY 13202, USA; ^3^Center for Computational Research, New York State Center of Excellence in Bioinformatics and Life Sciences, State University of New York (SUNY), Buffalo, NY 14260, USA

## Abstract

MicroRNAs (miRNAs) regulate gene expression posttranscriptionally. Although previous efforts have demonstrated the functional importance of target sites on miRNAs, little is known about the influence of the rest of 3′ untranslated regions (3′UTRs) of target genes on microRNA function. We conducted a genome-wide study and found that the entire 3′UTR sequences could also play important roles on miRNA function in addition to miRNA target sites. This was evidenced by the fact that human single nucleotide polymorphisms (SNPs) on both seed target region and the rest of 3′UTRs of miRNA target genes were under significantly stronger negative selection, when compared to non-miRNA target genes. We also discovered that the flanking nucleotides on both sides of miRNA target sites were subject to moderate strong selection. A local sequence region of ~67 nucleotides with symmetric structure is herein defined. Additionally, from gene expression analysis, we found that SNPs and miRNA target sites on target sequences may interactively affect gene expression.

## 1. Introduction

miRNAs are small noncoding RNAs of ~23 nucleotides. They are one of the major regulatory gene families playing important roles in almost every cellular process in animals, plants and viruses [1–3]. In animals, this includes regulation of developmental timing and signaling pathways, apoptosis, metabolism, myogenesis and cardiogenesis, brain development [1], and human pathologies [4–6]. 

It is believed that miRNAs mainly mediate gene regulation posttranscriptionally via translational repression and reduction of mRNA stability by forming miRNA-mRNA pairs to their target genes. In vertebrates, most miRNAs pair imperfectly with their target 3′UTRs, with a contiguous and perfect base pairing of the miRNA nucleotides 2–7 or 2–8 in the “seed” region. The miRNA seed target region on target gene, which is very important for target recognition, provides pairing specificity [7–9] for translational suppression. However, these seed matches are not always sufficient for repression. The degree of repression might also be related to other features on target 3′UTRs, including AU-rich nucleotide composition of 3′UTR [10] or AU-rich nucleotide composition near miRNA target sites [11], the location of miRNA target sites on 3′UTRs [11–13], the site accessibility in miRNA target recognition [14–16], and base pairing pattern outside seed target region [11].

SNPs are an abundant form of genome variation. Although most SNPs have little or no effect on gene regulation and protein activity, there are many circumstances where base changes in coding regions change the protein anthology and in noncoding regions affect the dynamics of gene expression through the influences of DNA and RNA structures. SNPs residing in miRNA genes can alter pri-miRNA processing and maturation [17, 18], affect miRNA-mRNA interaction, and decrease mature miRNA expression [19, 20], resulting in disease phenotype such as tumorigenesis [20] and esophageal cancer [21]. SNPs within miRNA target sites can modulate miRNA-mRNA interaction to create or destroy miRNA binding to target sites, resulting in new phenotypes [22–26]. There are also reports indicating that SNPs outside miRNA target sites can affect miRNA function. One recent finding [27] demonstrates that a polymorphism near miR-24 target site in the 3′UTR of human dihydrofolate reductase gene affects its expression by interfering with the miR-24 function, resulting in dihydrofolate reductase overexpression and methotrexate resistance.

Chen and Rajewsky found that SNP density in miRNA target genes was significantly lower in the region matching the 5′ end of miRNA than in the rest of the target site and that significant negative selection acted on a large class of computationally predicted conserved miRNA target sites [28]. These results were in agreement with the finding reported by Saunders et al. who discovered that the average SNP density in computationally predicted target sites was much lower than that in flanking regions [29]. All these results indicate that miRNA target sites play very important roles for miRNA function. However, little is known about the impact from the rest of target 3′UTR nucleotides on the functionality of miRNAs, as polymorphism outside miRNA target site can interfere with miRNA function [27]. In an attempt to gain insight into the functional importance of target 3′UTRs on miRNAs, we conducted studies to compare the difference of selective pressures for SNPs between miRNA and non-miRNA target 3′UTRs. Given the broad function of miRNAs, it is expected that miRNA target sequences, if they contribute to the functionality of miRNAs, would be under strong evolutionary selective pressures and therefore have less genetic variations, as compared to non-miRNA target sequences. We have found that SNPs on both seed target region and the rest of 3′UTRs of miRNA target genes were under stronger negative selection, suggesting the functional role of the 3′UTRs on miRNAs. We also found that other than the seed target region, a continuous nucleotide region surrounding the seed target region was subject to moderate strong selection, indicating more important roles on miRNA function for local sequences. Additionally, from correlation and gene expression data analyses, we found that SNPs on the target sequences may have functional roles related to miRNAs.

## 2. Methods

### 2.1. 3′UTRs of Human Genes and miRNA Data

We downloaded genomic coordinates and the length of 3′UTRs for all human genes (hg18 March 2006 assembly) with Reference Sequences (RefSeqs) from UCSC genome browser (http://genome.ucsc.edu/) and predicted miRNA target sites (hg18 March 2006 assembly) from TargetScan (http://www.targetscan.org/). These miRNA target data sets contain the human gene names and the genomic coordinates with the seed region for each individual miRNAs. In the case of a gene with alternative-splicing variants but the same 3′UTR sequence, one of the 3′UTRs was chosen. We obtained ~20170 3′UTRs for human RefSeqs, which represent ~18080 unique genes.

### 2.2. Compilation of SNP, Derived Allele Frequency (DAF), and Minor Allele Frequency (MAF) Data

We obtained human SNP data (dbSNP Build 130) with SNP IDs, genomic coordinates, and polymorphism types from UCSC genome browser (http://genome.ucsc.edu/). Insertion and deletion polymorphisms were discarded. Using genomic coordinates, we mapped SNPs located on 3′UTRs to the 20170 human RefSeqs. The data for DAF and MAF were obtained from three data tables SNPAncestralAllele, SNPAlleleFreq, and Allele (dbSNP Built 130). The SNPAncestralAllele table contains ancestral allele information and the SNPAlleleFreq table provides the allele frequencies for SNPs with both tables being organism specific (ftp://ftp.ncbi.nih.gov/snp/organisms/human_9606/database/). The allele table (ftp://ftp.ncbi.nih.gov/snp/database/shared_data/) contains allele information for all organisms. We first joined these three tables to get the frequency of each allele for SNPs located on the 3′UTRs of the 20170 human RefSeq genes, including the ancestor allele. We then computed for individual SNPs the DAF by removing the fraction of ancestor allele. For multiple alleles at a locus, the one with lowest allele frequency was selected as MAF. In the case of only one allele for a SNP, MAF is considered to be 0.

### 2.3. Gene Expression Data and Analysis of Expression Variation

Gene expression data from Stranger et al. [30] was used for the analyses of expression variation between miRNA and non-miRNA target genes with or without SNPs on the 3′UTRs. This dataset, which comes from illumina 6 × 2 Human gene expression arrays, contains gene expression profiling of Epstein-Barr virus-transformed lymphoblastoid cell lines of all 270 individuals genotyped in the HapMap Consortium. These individuals include 90 Yoruban individuals (YRI), 45 Japanese (JPT), 45 Han Chinese (CHB), and 90 Utah residents with ancestry from northern and western Europe (CEU). The gene expression data, which contains ~18980 unique RefSeqs, were normalized with quantile normalization across replicates and medial normalization across all individuals and therefore can be used for joint analyses for all populations or within each population. 

For each gene, the coefficient of variation (CV) of expression signals across all 270 individuals was computed, which is the ratio of the standard deviation to the mean expression signal. The CV difference between gene groups was then compared. These groups include (1) non-miRNA target genes without SNPs on their 3′UTRs; (2) non-miRNA target genes with SNPs on their 3′UTRs; (3) miRNA target genes without SNPs on their 3′UTRs; (4) miRNA target genes with SNPs on their 3′UTRs; (5) miRNA target genes with SNPs in the seed target region; (6) miRNA target genes with SNPs in the rest but seed target region; (7) miRNA target genes with SNPs in the defined SNP density window region; (8) miRNA target genes with SNPs in the rest but defined SNP density window region. Two-way ANOVA was also employed to compare the effect on CVs between miRNA and non-miRNA target genes, target genes with SNPs and without SNPs, and the interaction between SNPs and miRNAs. The GLM is: CV = miRNA + SNP + miRNA × SNP + error. 

### 2.4. Rank Test

Rank test was employed to estimate correlation between SNP density and miRNA target site density. Genes were first ranked by either the density of miRNA target sites or the density of SNPs, resulting in 2 datasets with either ordered density of miRNA target sites or ordered SNP density. Genes in each of the two resulting datasets were then separated into 5 equal number groups, which were represented as [1,20], (21,40], (41,60], (61,80], and (81,100] in standard interval notation with increasing values. Comparison analyses for the paired values of miRNA target site density and SNP density were performed within each of the 5 gene groups. 

### 2.5. Significance Tests

To assess the statistical significance for the window sizes of SNP density (67 nucleotides), DAF (28 nucleotides), and MAF (26 nucleotides) surrounding miRNA target sites, we performed tests to estimate the occurrences of such window sizes using randomly selected SNP density, DAF, or MAF from 3′UTRs of miRNA target genes. The same number of nucleotides for each window size was randomly sampled without replacement from 3′UTRs of all miRNA target genes with SNPs and then used to compare the values of SNP density, MAF, or DAF to those from non-miRNA target genes. The analyses for each window size were performed 100,000 times, and the *P* values were obtained by computing the number of times out of 100,000 simulated windows have larger SNP density, or lower DAF/MAF as compared to those from non-miRNA target genes.

## 3. Results

### 3.1. The Impact of 3′UTRs on miRNA Function Inferred from SNPs

SNPs are the most abundant type of genetic polymorphisms in genomes. While functional SNPs have the ability to influence the structure of DNA, RNA, or proteins, they are believed to contain the signatures of evolutionary selection for the functional elements on the genome. We chose to compare the SNP density between 3′UTRs of miRNA target genes and non-miRNA target genes, which should be able to reveal whether or not miRNA target sequences are under strong evolutionary selective pressures due to miRNAs and, therefore, the functional importance of target sequences on miRNAs. 

The SNP density from non-miRNA target genes and miRNA target genes is listed in Table 1, in which the number of SNPs and nucleotides from the 3′UTRs of miRNA target genes and non-miRNA target genes are also listed. Out of 8,529,061 nucleotides from the 3′UTRs of non-miRNA target genes, 47,230 of them display SNP features with a density of 5.54/kb. By contrast, the SNP density on the 3′UTRs of miRNA target genes is much lower. Of the total 13,795,877 nucleotides, 54,890 display SNP features (3.99/kb), indicating that SNPs occur less frequently in the 3′UTRs of miRNA target genes than non-miRNA target genes (Fisher's exact test *P* = 1.5 × 10^−154^). We were curious to know if the length of 3′UTRs from individual genes would have impact on the occurrence of SNPs and performed further analysis to calibrate SNP density from individual genes. We first computed SNP density on each individual gene and then compared SNP density difference at gene level between miRNA target genes and non-miRNA target genes. In agreement with the above finding, the results indicate that the median SNP density of individual miRNA target genes (3.8 SNPs/1000 nucleotides) is significantly (Wilcoxon rank sum test *P* = 4.1 × 10^−126^) lower than that of individual non-miRNA target genes (5.2 SNPs/1000 nucleotides). 

**Figure 1:**
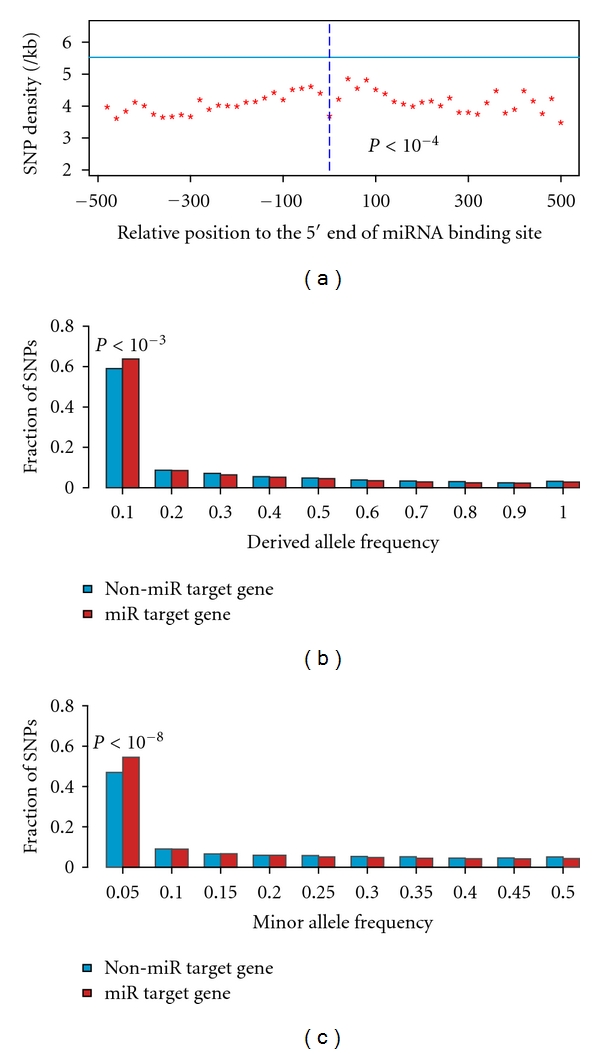
Distribution of SNP density, DAF, MAF on the 3′UTRs of miRNA and non-miRNA target genes. (a) The distribution of SNP density for 500 nucleotides both upstream and downstream of miRNA target sites. Each asterisk represents the average SNP density for a 20 nucleotide window size. The average SNP density from non-miRNA target genes is depicted in solid lines. Dotted lines indicate the 5′ end of miRNA target sites. *P* values are from Fisher's exact tests comparing between each bin and those from non-miRNA target genes. (b) SNPs for both miRNA and non-miRNA target genes were separated into 10 bins with DAF increment of 0.1, and the fraction of SNPs in each bin was computed. (c) SNPs for both miRNA and non-miRNA target genes were separated into 10 bins with MAF increment of 0.05, and the fraction of SNPs in each bin was computed.

We next performed analysis to investigate the distribution of SNP density surrounding miRNA target sites. In this analysis, we computed the occurrence of SNPs at each nucleotide location for nucleotides both upstream and downstream of the 5′ end of miRNA target site. In the case of multiple miRNA target sites on a gene's 3′UTR, we extended the computing to the middle nucleotide location of two adjacent miRNA target sites. We then separated them to bins, each covering 20 nucleotide locations, and computed SNP density for each bin. The results indicate that SNP density for each bin on both sides of miRNA target sites is significantly lower (Fisher's exact test *P* between 3.5 × 10^−5^ and 9.6 × 10^−105^) than those from the 3′UTRs of non-miRNA target genes, as shown in Figure 1(a), where SNP density for 500 nucleotides both upstream and downstream of miRNA target site is depicted. This result reveals that not only miRNA target sites but also the rest of target 3′UTRs could play important roles on miRNA function. 

To further confirm the above findings, we performed analysis to compare DAF between 3′UTRs of miRNA and non-miRNA target genes, since an excess of rare derived alleles is a signature of negative selection due to the sensitivity of SNP density to mutation rate heterogeneity [28]. Based on the frequency in HapMap data [31], SNPs for both miRNA and non-miRNA target genes were first separated into 10 bins with DAF increment of 0.1, and the fraction of SNPs in each bin was then computed. The results indicate that DAF distribution of SNPs from both miRNA and non-miRNA target genes is heavily skewed toward rare derived alleles as shown in Figure 1(b), where majority of SNPs have DAF ≤ 0.1, indicating that SNPs on 3′UTRs were under strong negative selection. Further analyses demonstrate that 64% SNPs from miRNA target genes are rare derived alleles (DAF ≤ 0.1), when compared to 59% SNPs from non-miRNA target genes (Fisher's exact test *P* < 10^−3^). We also investigated the SNP distribution for minor alleles, which harbor deleterious mutations limited by natural selection. MAF, therefore, provides further evidence for negative selection. Similar to DAF results, MAF distribution of SNPs from both miRNA and non-miRNA target genes is heavily skewed toward rare minor alleles (MAF ≤ 0.05) as shown in Figure 1(c), in which SNPs from miRNA target genes have a higher fraction (55%) of rare minor alleles (MAF ≤ 0.05), as compared to SNPs from non-miRNA target genes (47%; Fisher's exact test *P* < 10^−8^). 

Taken together, our results show that miRNA target 3′UTRs are under stronger negative selection, most likely due to the presence of functional miRNA targets. These results also demonstrate that the low SNP density on miRNA target 3′UTRs, as compared to those from non-miRNA target genes, are not due to the potential source of bias from the prediction of miRNA target sites with preferential cross-species conservation. This was supported by the findings from both the distributions of SNP density and DAF.

### 3.2. The Importance of Target Local Sequences on miRNAs

Previous studies [28, 29] showed that miRNA seed region was subject to stronger negative selection, when compared to nucleotides in other region of the genome, suggesting that the seed region is critical for miRNA function. Other than the seed region, local environment such AU content on target 3′UTRs [11] could also affect the functionality of miRNAs. We were curious to know if there exists any nucleotide region surrounding miRNA target sites that could have great impact on miRNA function. 

Accordingly, we performed analysis to investigate SNP density, the distribution of SNPs for minor alleles and the distribution of SNPs for derived alleles surrounding miRNA target sites. As mentioned above, we computed the occurrence of SNPs, SNPs of minor alleles, and SNPs of derived alleles at each nucleotide location on both sides of the 5′ end of miRNA target sites. Since the rare derived alleles and rare minor allele, which constitute a large part of SNPs, are signatures for strong negative selection, we employed DAF ≤ 0.1 and MAF ≤ 0.05 for the analyses of their distributions surrounding miRNA target sites. 

**Figure 2:**
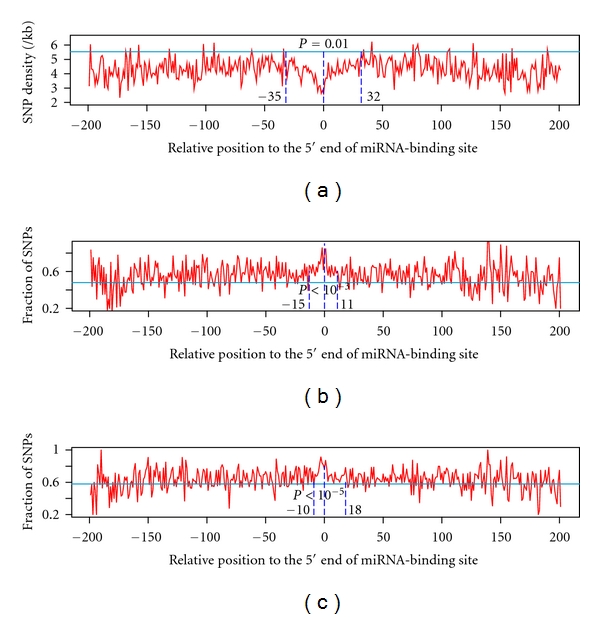
Distribution of SNP density, DAF, and MAF surrounding miRNA target sites. The SNP density (a), the fraction of SNPs for minor alleles (b) and derived alleles (c) obtained from all miRNA target genes having SNPs for the 200 nucleotides on both sides of the 5′ end of miRNA target sites are shown. The average SNP density and fraction of minor and derived alleles from non-miRNA target genes are depicted in solid lines. Dotted lines indicate the 5′ end of miRNA target sites (middle), upstream bound (left), and downstream bound (right). Also shown are the *P* values for the occurrence of the window sizes from simulation tests.

The results are shown in Figure 2, where SNP density, the fraction of SNPs from derived and minor alleles obtained from all miRNA target genes are depicted for the 200 nucleotides on both sides of region matching the 5′ end of the miRNA. We found that a ~67 continuous nucleotide region surrounding miRNA target sites displayed lower SNP density with 32 nucleotides on the right side and 35 nucleotides on the left side, as compared to the average SNP density from non-miRNA target genes (Figure 2(a)). The occurrence of this window size with lower SNP density is statistically significant (*P* = 0.01) based on 100,000 datasets, each having 67 nucleotides randomly sampled from miRNA target 3′UTRs. Similar results were also observed for the distribution of MAF and DAF although the window sizes surrounding miRNA target sites are smaller. While 26 nucleotides (*P* < 10^−3^) display higher SNP frequency for minor alleles surrounding miRNA target sites with 11 nucleotides on the right side and 15 nucleotides on the left side (Figure 2(b)), the DAF window size is 28 nucleotides (*P* < 10^−5^) with 18 on the right side and 10 on the left side (Figure 2(c)). To confirm the above findings, we next performed analyses with a smaller window size of 8 nucleotides. Starting from miRNA seed target region, we moved this smaller window 2 bases each time toward either 3′ or 5′ end and computed for each case the statistical significance based on randomly sampled datasets from miRNA target 3′UTRs. Significant lower SNP density (*P* < 10^−2^) was observed for all cases when this smaller window size was within the defined 67 nucleotide window. These results suggest that local environment with a certain size of nucleotides on both sides of miRNA target site is most likely needed for miRNAs to function properly.

### 3.3. Comparison of SNP Density, DAF, and MAF for Different Regions of Target Sequences

**Figure 3:**
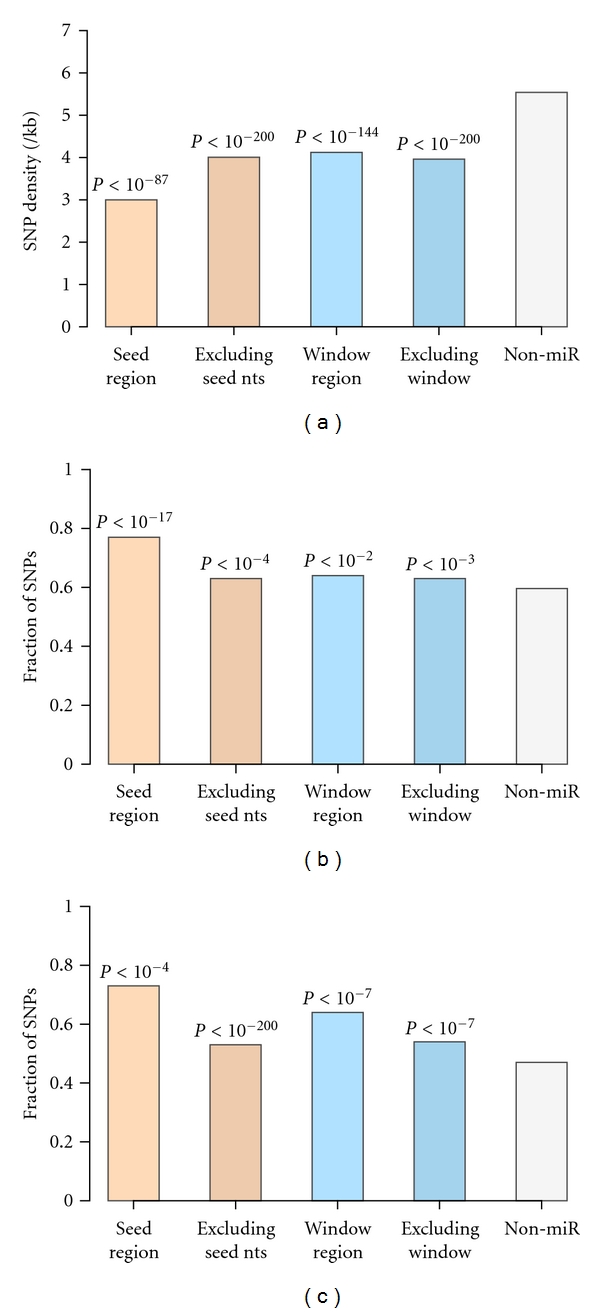
Comparison of SNP density, DAF, and MAF between different regions of miRNA target genes and non-miRNA target genes. SNPs on miRNA target sequences were separated into (1) seed target region: region matching the 5′ end of the miRNA; (2) excluding seed target region: all the rest of 3′UTRs without seed target region; (3) window regions: regions surrounding the 5′ end of the miRNA target site defined in this study, 67 nucleotides for SNP density, 28 nucleotides for DAF, and 26 nucleotides for MAF; (4) excluding the window regions: all the rest of 3′UTRs without the corresponding window regions. SNPs from these defined regions were compared to the SNPs from non-miRNA target genes. (a) All 4 regions from miRNA target sequences have lower SNP density than non-miRNA target genes. (b) All 4 regions from miRNA target sequences have higher fraction of DAF than non-miRNA target genes. (c) All 4 regions from miRNA target sequences have higher fraction of MAF than non-miRNA target genes. Also shown are the Wilcoxon rank sum test *P-*values comparing to non-miRNA target genes.

To investigate the influence on miRNA function from different target sequences, we separated SNPs into 2 groups with one group from window region defined in this study and the other group from the rest of target 3′UTRs. We also separated SNPs into groups from seed target region and from the rest of target 3′UTRs. We then computed SNP density as well as SNP frequency for minor alleles and derived alleles for each group. The findings show that SNP density and SNP frequency for derived alleles and minor alleles from all 4 groups are significantly different (*P* between 10^−3^ and 10^−200^) from those of non-miRNA target genes (Figure 3). While the seed target region has the lowest SNP density and largest fraction of DAF and MAF, which is in agreement with previous reports [28, 29], the rest 3 groups have similar patterns of lower SNP density and higher SNP frequency for derived and minor alleles, as compared to non-miRNA target genes. These results further exemplify that other than seed target region, the rest of the target 3′UTRs may play important roles on miRNA function. 

### 3.4. SNPs on miRNA Target Sequences Are Most Likely to Have Functional Roles Related to miRNAs

**Figure 4:**
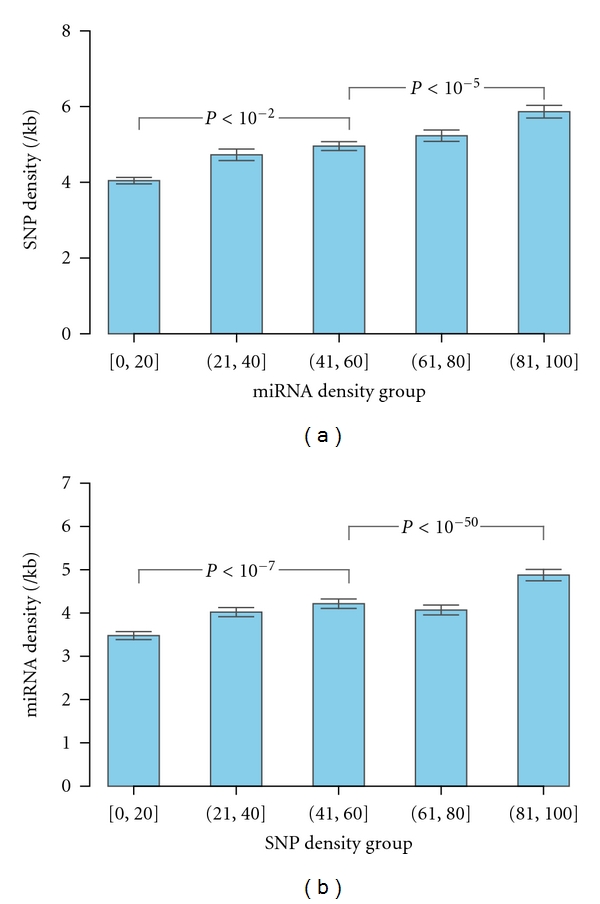
Relationship between miRNA target site density and SNP density. (a) Variation of SNP density for 5 miRNA target site density groups with increasing density of target sites. The degree of the increasing trend for SNP density along with the increasing density of miRNA target sites was estimated by Wilcoxon rank sum tests and depicted in *P* values. (b) Variation of miRNA target site density for 5 SNP groups with increasing density. The degree of the increasing trend for miRNA target site density along with the increasing density of SNPs was estimated by Wilcoxon rank sum tests and depicted in *P* values. Error bars indicate standard errors.

Since miRNAs play important biological roles for cells, one would expect that miRNA target sequences should have not only lower SNP density than that of non-miRNA target sequences but also a negative correlation between the miRNA target site density and SNP density. Our findings, however, indicate that miRNA target site density and SNP density are positively correlated (Spearman's rank correlation rho: 0.13; *P*: 2.2 × 10^−18^). To further confirm our findings, we employed a rank test to assess the density relationship between miRNAs and SNPs. In agreement with the correlation result, as the density of miRNA target sites increases, the average SNP density increases as shown in Figure 4(a), where the distribution of the average SNP density for 5 miRNA target site density groups is depicted. Statistical analyses show that the average SNP density in higher miRNA target site density groups is significantly larger than those in lower miRNA target site density groups (one-side Wilcoxon rank sum test *P* < 10^−2^). This observation is also true for the SNP density ranked analysis as shown in Figure 4(b), where the average miRNA target site density increases along with the increasing of SNP density, with significant differences of miRNA target site density (one-side Wilcoxon rank sum test *P* < 10^−7^) between low, medium, and high SNP density groups.

These results suggest that SNPs in miRNA target sequences could have functional roles related to miRNAs. Therefore, it is expected that some of these SNPs came from very recent selection in favor of new alleles that were better suited to the environments. To test if SNPs from miRNA target sequences were also under stronger positive selection, we compared the difference of integrated haplotype scores (iHS), which measure the recent positive selection for a given haplotype [32], for SNPs between miRNA and non-miRNA target genes. Using values of ∣iHS∣ from 3 haplotypes of 90 Yoruban individuals (YRI), 90 Asian individuals (ASI), and 90 Utah residents with ancestry from Northern and Western Europe (CEU) in the HapMap Consortium [31], we compared within each haplotype the ∣iHS∣ for SNPs between miRNA and non-miRNA target genes as well as seed target region and density window regions defined in this study. The results indicate that SNPs from miRNA target genes were under stronger positive selection in CEU and ASI, as compared to SNPs from non-miRNA target genes, although statistical significances were not observed (data not shown). It is worthy to note that the strongest positive selection were observed in the seed target region and density window region defined in this study, for which all 3 haplotypes display larger ∣iHS∣, as compared to those for SNPs from non-miRNA target genes. 

**Figure 5:**
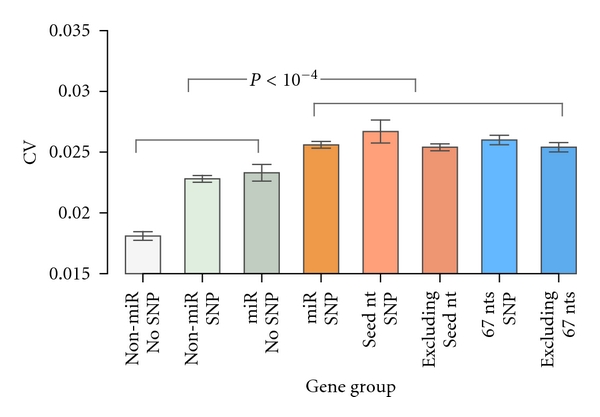
Comparison of CVs between different gene groups. Genes with both miRNA target sites and SNPs have significant larger CVs (one-side Wilcoxon rank sum test, *P* < 10^−4^). Gene groups include non-miR no SNP: non-miRNA target genes without SNPs on their 3′UTRs; non-miRNA SNP: non-miRNA target genes with SNPs on their 3′UTRs; miR no SNP: miRNA target genes without SNPs on their 3′UTRs; miR SNP: miRNA target genes with SNPs on their 3′UTRs; seed nt SNP: miRNA target genes with SNPs in the seed target region; excluding seed nt: miRNA target genes with SNPs in the rest but seed target region; 67 nts SNP: miRNA target genes with SNPs in the SNP density window region defined in this study; excluding 67 nts: miRNA target genes with SNPs in the rest but defined density window region.

As a first step to investigate the relationship between SNPs and miRNAs on gene regulation, we extended our analysis to see if they act interactively using data from Stranger et al. [30]. For each gene, we first obtained the CV across 270 individuals from the HapMap Consortium [31] and then compared the CV differences between different gene groups (see Section 2). Since SNPs can affect miRNAs to either increase or decrease the expression of a gene, the use of CV, therefore, provides the most unambiguous measurement of gene expression variability [33] due to SNPs or miRNAs. The results indicate that miRNA target genes with SNPs have significant larger CVs (one-side Wilcoxon rank sum test, *P* < 10^−4^; Figure 5). We also performed permutation tests to randomly get sets of genes with the same size of miRNA target genes with SNPs from either all genes or genes with SNPs. The median CVs from these selected genes were computed and compared to that of miRNA target genes with SNPs. Significantly higher CVs (*P* < 10^−5^) were also observed in miRNA target genes with SNPs, when compared to the random sets of genes. This result is not surprising, as both miRNAs and SNPs could influence gene expression, which was further illustrated by a two-way ANOVA analysis showing that CVs for miRNA target genes are significantly larger (*P* < 10^−15^) than CVs for non-miRNA target genes and CVs for genes with SNPs are significantly larger (*P* < 10^−10^) than CVs for genes without SNPs. It is important to note that there exists interacting effect on CVs between miRNAs and SNPs (*P* = 0.03). These results indicate that SNPs on target sequences may interactively act with miRNA to affect the variability of gene expression.

## 4. Discussion

miRNAs regulate gene expression posttranscriptionally by base-pairing to target mRNAs in 3′UTRs. Previous study indicated that 3′UTR length of miRNA target genes are significantly longer than those in non-miRNA target genes [34], suggesting that 3′UTRs play very important roles for miRNA function. In fact, early studies indicated that different features of miRNA target sequences could influence the repression of miRNAs on gene expression [22–26]. In this study, we conducted studies to gain new insight into the influence of target 3′UTRs on miRNA function by the use of human SNP data. 

The rationale behind this study is that if miRNAs are functional and target sequences influence their function, the SNPs on these sequences would be under stronger purifying selection when compared to those of non-miRNA target sequences. The advantage of comparing to the 3′UTRs of non-miRNA target genes, rather than the genome sequences or conserved sequences [28], is because the 3′UTRs, other than miRNA target sites, harbor target sites for other important regulatory elements. These regulatory elements include Selenocysteine insertion sequence, UNR-binding sites, Polyadenylation signal, Mos polyadenylatin response element, and K-boxes, Brd-boxes, and GY-boxes [35], which are known to play crucial roles in posttranscriptional regulation of gene expression such as translation, subcellular localization, and stability of the mRNA. The comparison results from using the 3′UTRs of non-miRNA target genes are, therefore, most likely to reflect the difference due to the functional elements of miRNAs. 

It is worthy to note that the prediction of miRNA targets depends on conservation information of 3′UTRs, SNPs on the 3′UTRs of miRNA target genes could, therefore, have lower density than those from non-miRNA target genes because of the potential source of bias from the prediction of miRNA target sites with preferential cross-species conservation. This concern was addressed by both the distribution of SNP density and the DAF analyses. The former demonstrated lower SNP density not only for the miRNA target sites but also the rest of 3′UTR sequences, when compared to those from the 3′UTRs of non-miRNA target genes. The latter compared the fraction of rare derived alleles between miRNA target genes and non-miRNA target genes, which was normalized by each target gene groups. 

We observed significant difference of SNP density between 3′UTRs of miRNA target genes and non-miRNA target genes, suggesting that miRNA target sequences in general are subject to stronger purifying selection. It is important to note that majority of SNPs accumulating overtime are neutral with no harmful or beneficial effect on human. These SNPs, which occur at a steady rate, can greatly influence the comparison results. Therefore, we employed both DAF and MAF to further confirm our findings. Whereas an excess of rare derived alleles is a signature of negative selection [28], minor alleles are most likely to harbor deleterious mutations that were subject to negative selection [36]. The findings that both miRNA and non-miRNA target genes have excess rare derived and minor alleles is an indicative of strong negative selection on the 3′UTRs of both miRNA and non-miRNA target genes, indicating the validity of using the 3′UTR of non-miRNA target genes as references for comparisons. The fraction of both rare derived alleles (DAF ≤ 0.1) and rare minor alleles (MAF ≤ 0.05) from miRNA target genes are significantly larger than those from non-miRNA target genes, presenting very strong evidence for a stronger purifying selection on SNPs from miRNA target genes. 

One of the purposes of this study is to find if there exists any nucleotide region surrounding miRNA target sites that could have great impact on miRNA function. Based on SNP density, we found that a local region of about ~67 nucleotides is most likely to play a critical role in miRNA function. This local region contains similar number of nucleotides on both sides of the seed target region. Whereas most of the nucleotides upstream of the seed target region might pair with the rest of the ~23 miRNA sequences, the downstream flanking sequences are located outside miRNA target sites, raising an interesting question as to whether a symmetric structure is needed for miRNA seed efficacy. It is important to note that the seed target region display the lowest SNP density, which is in agreement with other's findings [28]. We did not however find any significant difference between the upstream and downstream sequences for SNP densities in the window region. These findings were exemplified by both MAF and DAF analyses, and although results from MAF and DAF display smaller local sequence region than SNP density, they nevertheless show similar symmetric structures like SNP density with up to 10 to 18 nucleotides downstream of miRNA target sites. 

One of the interesting findings from this study is that SNP density and miRNA target site density on miRNA target genes are positively correlated, suggesting that SNPs may be functionally related to miRNA target sites. It is therefore expected that some of the SNPs that were first under purifying selection might be also subject to positive selection, resulting in new genotypes that were better suited to the environments. It is known that advantageous substitutions are rare and are usually overwhelmed by the large number of neutral substitutions. We, nevertheless, found stronger positive selection for SNPs from miRNA target genes than those from non-miRNA target genes, although no statistical significance was observed, indicating that some of the SNPs on miRNA target sequences might have new functional roles other than affecting miRNA-mRNA interactions. Further evidence comes from the variation analysis for gene expression, in which SNPs and miRNAs could interactively affect the variability of gene expression.

## Figures and Tables

**Table 1:** Comparison of SNP density on 3′UTRs between miRNA target genes and non-miRNA target genes.

	No. SNPs	No. nucleotides	Density (SNP/kb)	*P value
Non-miRNA target gene with SNPs	47230	8529061	5.538	
miRNA target gene with SNPs	54980	13795877	3.985	1.50E-154

*From Fisher's exact test.
